# Chemical characterization of Saudi propolis and its antiparasitic and anticancer properties

**DOI:** 10.1038/s41598-021-84717-5

**Published:** 2021-03-08

**Authors:** Samyah Alanazi, Naif Alenzi, Fouza Alenazi, Hajera Tabassum, David Watson

**Affiliations:** 1 Department of Clinical Laboratory Sciences, College of Applied Medical Sciences, King Saud University, Riyadh, Saudi Arabia; 2 Research and Laboratories Sector, National Drug and Cosmetic Control Laboratories (NDCCL), Saudi Food and Drug Authority, Riyadh, Saudi Arabia; 3 Strathclyde Institute of Pharmacy and Biomedical Sciences, University of Strathclyde, Glasgow, UK

**Keywords:** Biochemistry, Biological techniques, Cancer, Plant sciences, Health care, Medical research, Chemistry

## Abstract

Propolis, is a gummy material produced by honey bees from different parts of plants and is enriched with varied biological active compounds like flavonoids, phenolics and phenolic acids with wide applicability in the food, pharmaceutical and cosmetics industries. The current report is focused on the characterisation of propolis collected from Asir region, South-west of Saudi Arabia and its effect on *Trypanosoma brucei* (the causative organism of African sleeping sickness) and cytotoxic effect against U937 human leukemia cells. The Chemical composition and spectral characteristics of Saudi propolis was studied by Liquid Chromatography Mass Spectrometry (LC–MS) and High-performance liquid chromatography–evaporative light scattering detector (HPLC–ELSD).The two main active compounds isolated from Saudi propolis via column chromatography and size exclusion chromatography were fisetinidol and ferulic acid. High resolution electrospray ionization–mass spectrophotometer (HRESI–MS) and nuclear magnetic resonance (NMR) were used to elucidate the structures of the isolated compounds. All crudes extracts, fractions as well as isolated compounds were subjected for biological testing against *Trypanosoma brucei* (S427 WT), and their cytotoxicity against U937 human leukemia cells. Amongst the various samples investigated, S-6 fraction demonstrated highest anti-trypanosomal activity at 2.4 µg/ml MIC followed by fisetinidol at 4.7 µg/ml reflecting that the anti-trypanosomal activity is attributable to the presence of fisetinidol in the fraction. Similarly, all the tested samples exhibited cytotoxicity with an IC50 > 60 µg/ml. S-6 fractions exhibited highest cytotoxic activity against U937 cells with an IC50 of 58.7 µg/ml followed by ferulic acid with an IC50 87.7 µg/ml indicating that the cytotoxic effect of propolis might be due to the presence of ferulic acid. In conclusion, the biological activity of propolis could be attributed to the synergistic action of the two active compounds-ferulic acid and fisetinidol. The data obtained in the study is thus indicative of the role of propolis as potential anti-trypanosomal and anticancer agent for effective cancer therapy.

## Introduction

Propolis is a natural gummy, and resinous substance with a complex and diverse composition comprising mainly beeswax and secondary metabolites from plants. Propolis or bee glue is produced by honeybees (*Apis mellifera* L.) from plant-derived materials during the process of sterilizing the hive environment^1^, thus ensuring the bee community’s health. In Saudi Arabia, the mountainous area in the south-west of the country, including Asir, is considered ideal for honey collection^2^ due to the conditions and vegetation (moderate weather during summer in addition to an abundance of trees and flowery shrubs) that are conducive to the bee species of *Apis mellifera jemenitica*^3^. Phytochemical data on Saudi Arabian propolis is scarce. It is speculated that the propolis production involves partial digestion or mixing with saliva of the materials gathered from plant bark, buds and flowers^4^, however, there is no positive evidence of such chemical changes^1^. Bees colonies that produce substantial quantities of propolis have been reported to be cleaner, achieve greater honey production, have a highly viable brood, and longer living worker bees^5^.

Propolis has been used as a popular traditional medicine since ancient times throughout the world^6^. It is currently used as alternative medicine and in health foods. The chemical composition of propolis varies by geographical location, botanical source, and bee species^7^. Until 2000, over 300 chemical components belonging to the flavonoids, terpenes, and phenols have been identified in propolis. The characteristic constituents of propolis in the temperate region are flavonoids such as chrysin, galangin, pinocembrin, and pinobanksin. Caffeic acid phenethyl ester is a major constituent of temperate propolis with broad biological activities. Brazilian green propolis primarily contains prenylated phenylpropanoids and diterpenes. Propolis from the Pacific region contains geranyl flavanones, which are also found in propolis from African regions^8^.

Propolis is extensively employed in preventing and treating colds, wounds, and ulcers, rheumatism, sprains, heart disease, diabetes, and dental caries due to its biological effects against cancer^9^, inflammation, and oxidants^10^, microbes^11^, and cell growth^12^. Based on the age and origin, propolis displays different colors yellow, dark brown, or even transparent^13^. At temperatures in the range of 25–45 °C, propolis typically begins to soften, becomes malleable, and highly sticky. Conversely, at temperatures below 15 °C, propolis becomes hard and brittle, and after it is frozen, it maintains its brittleness even at high temperatures. The usual melting point for propolis is 60–70 °C, although it may be up to 100 °C for some samples. Ethanol, ether, glycerol, and water are the main solvents employed for extracting propolis for commercial purposes; other solvents are also available^14^. Ethanol is mostly used to obtain low wax propolis extracts rich in biologically active compounds. The chemical composition of propolis has attracted interest due to its broad use in modern herbal medicine. Studies from different countries have shown the antimicrobial^15,16^, anti-inflammatory^17^, cytotoxic^18,19^, antiparasitic properties^20^, immunomodulatory^21,22^, and anti-leishmanicidal effects of different propolis extracts from different sources^23,24^. However, data on the anticancer and anti-trypanosomal activity of Saudi propolis is scarce. In view of the above, the present study was undertaken to chemically characterize the compounds in Saudi propolis and evaluate its anti-trypanosomal and anticancer properties.

## Results

### Profiling of crude Saudi propolis with LC and HPLC–UV–ELSD

**Figure 1:**
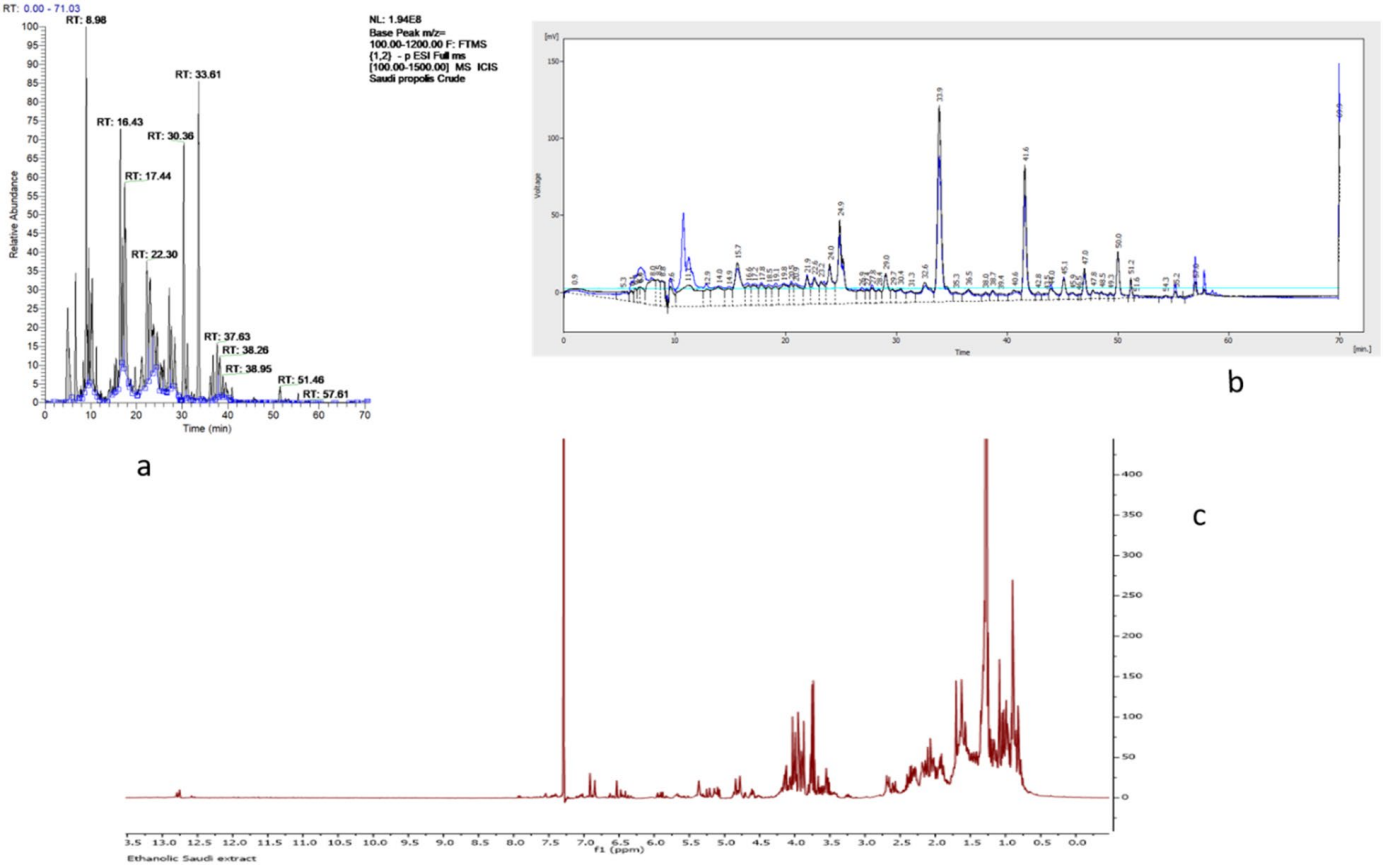
Chemical profile of crude Propolis extract (**a**) LC–MS Chromatogram of ethanol-based crude extract, (**b**) HPLC–ELSD profile of ethanolic crude Propolis extract and (**c**) ^1^H NMR spectrum of ethanol-based Saudi propolis extract in CDCl_3._

Crude propolis extract characterized using liquid chromatography–mass spectrometry (LC–MS) is shown in Fig. 1a. The LC–MS results detail most constituents and the nature of the constituents in the crude ethanolic extract. LC–MS profiling demonstrated the occurrence of flavonoids and other phenolics in the ethanol-based crude extract of propolis. The chromatograms of the crude samples run on HPLC–UV–ELSD (Fig. 1b) show a wide diversity in the chemical composition of propolis samples from Saudi Arabia.

The HPLC–UV–ELSD profile of the crude sample confirmed the occurrence of compounds absorbing ultraviolet (UV) light, such as flavonoids and phenolic compounds. Compounds lacking chromophores, including some terpenoids, were also identified; their intensities were low. Considerable complexity was exhibited by the LC–MS chromatogram of the crude sample, revealing multiple peaks of varying intensities. The flavonoids and phenolics were also confirmed as the dominant constituents by the ^1^H NMR spectra (Fig. 3c). In addition, terpenoids and fatty acid compounds were highlighted by a couple of signals captured by the NMR of the crude sample; their intensities were not as high as the flavonoids and phenolics.

### Isolation and characterization of pure compounds

**Figure 2:**
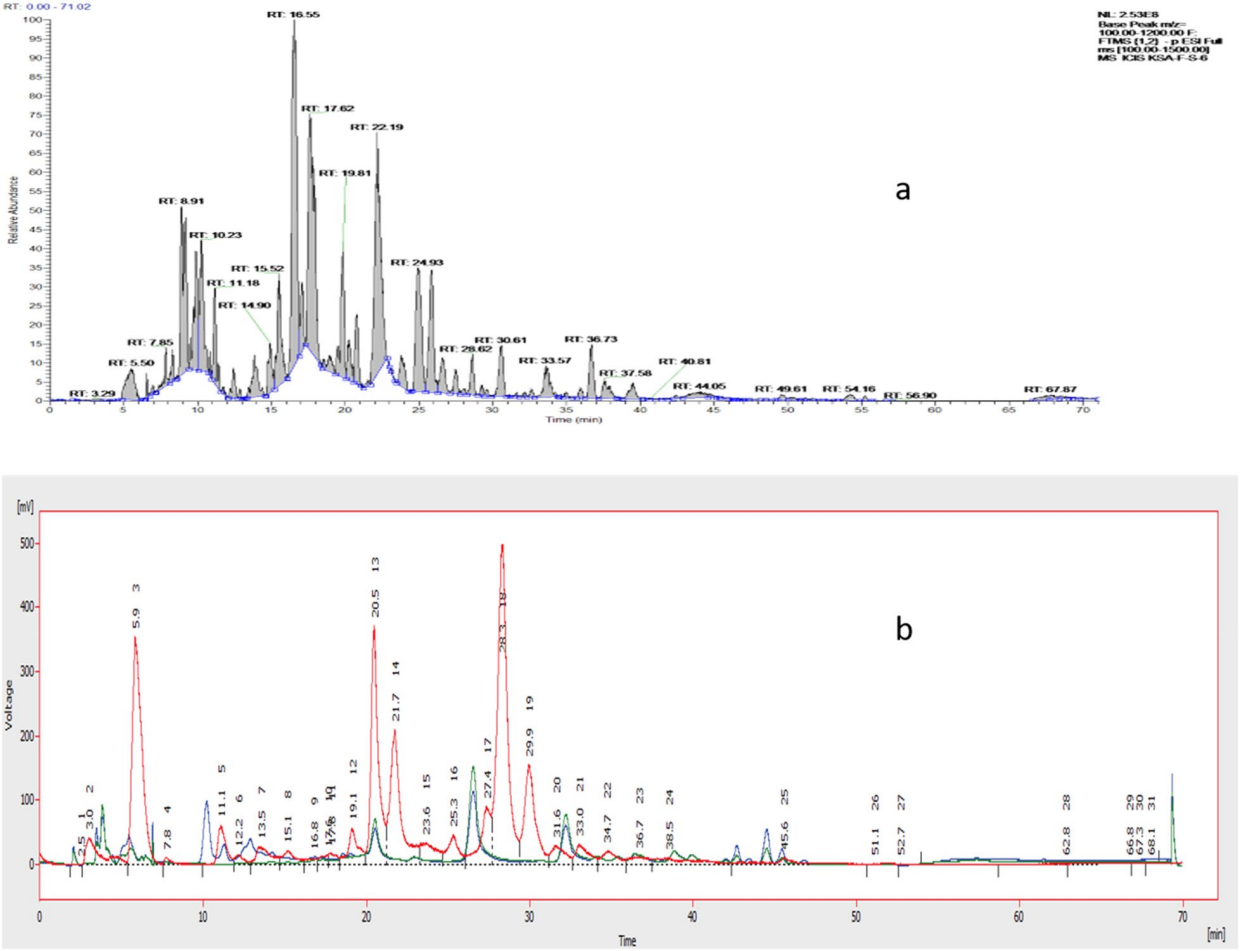
LC–MS Chromatogram of Saudi’s crude propolis fraction (S-6) using (**a**) ESI; (**b**) ESD (compounds were mostly UV-absorbing, that could be flavonoids or phenolic with retention times of 40 and 60 min (red and blue spectra corresponds to flavanoids detected at 290 and 320 nm respectively); Terpenoids or fats or any other compounds without chromophores (green trace) were also detected but with low intensities.

Column chromatography (CC) and size exclusion chromatography (SEC) were used to separate and purify propolis compounds. On CC, 28 fractions of the ethanol-based extract of propolis were obtained. The chromatographic characteristics were delineated via thin-layer chromatography using a suitable solvent system. The application of LC–MS and NMR permitted the identification of different components and allowed the combination of similar fractions. Similar fractions were combined to yield 10 pooled fractions. LC–MS and HPLC–UV–ELSD analysis revealed that the most abundant fraction, S-6, contained compounds with varied compositions, as shown in Table 1 and Fig. 2. The chromatogram view of Saudi’s fraction (S-6) on the ELSD showed that it contained mostly compounds with UV-absorbing activity, that could be flavonoids or phenols at retention times of 40 and 60 min (Fig. 2b). Terpenoids or fats, or any other compounds without chromophores were detected but at low intensities. Further, 475 mg of the fraction (S-6) from the CC was subjected to SEC, yielding 23 sub-fractions (S-6-1 to S-6-23), which led to the acquisition of two pure compounds (S-6-7 and S-6-13).

**Table 1:** Chemical profile of S-6 fraction by reverse-phase LC–MS.

Peak no	Retention time (min)	[M–H]–	Chemical formula	Delta (ppm)	Intensity
1	6.7	193.05	C_10_H_9_O_4_	− 0.011	E 6
2	9.04	273.08	C_15_H_13_O_5_	1.33	E 7
3	9.88	545.15	C_30_H_25_O_10_	1.357	E 7
4	15.57	405.08	C_19_H_17_O_10_	0.247	E 7
5	16.31	333.21	C_20_H_29_O_4_	1.252	E 7
6	19.66	335.22	C_20_H_31_O_4_	1.185	E 7
7	20.13	373.09	C_19_H_17_O_8_	1.472	E 7
8	22.17	419.1	C_20_H_19_O_10_	0.334	E 8
9	33.55	325.14	C_20_H_21_O_4_	1.315	E 7
10	36.64	317.21	C_20_H_29_O_3_	1.551	E 7

### Characterization of S-6-7 as fisetinidol

**Figure 3:**
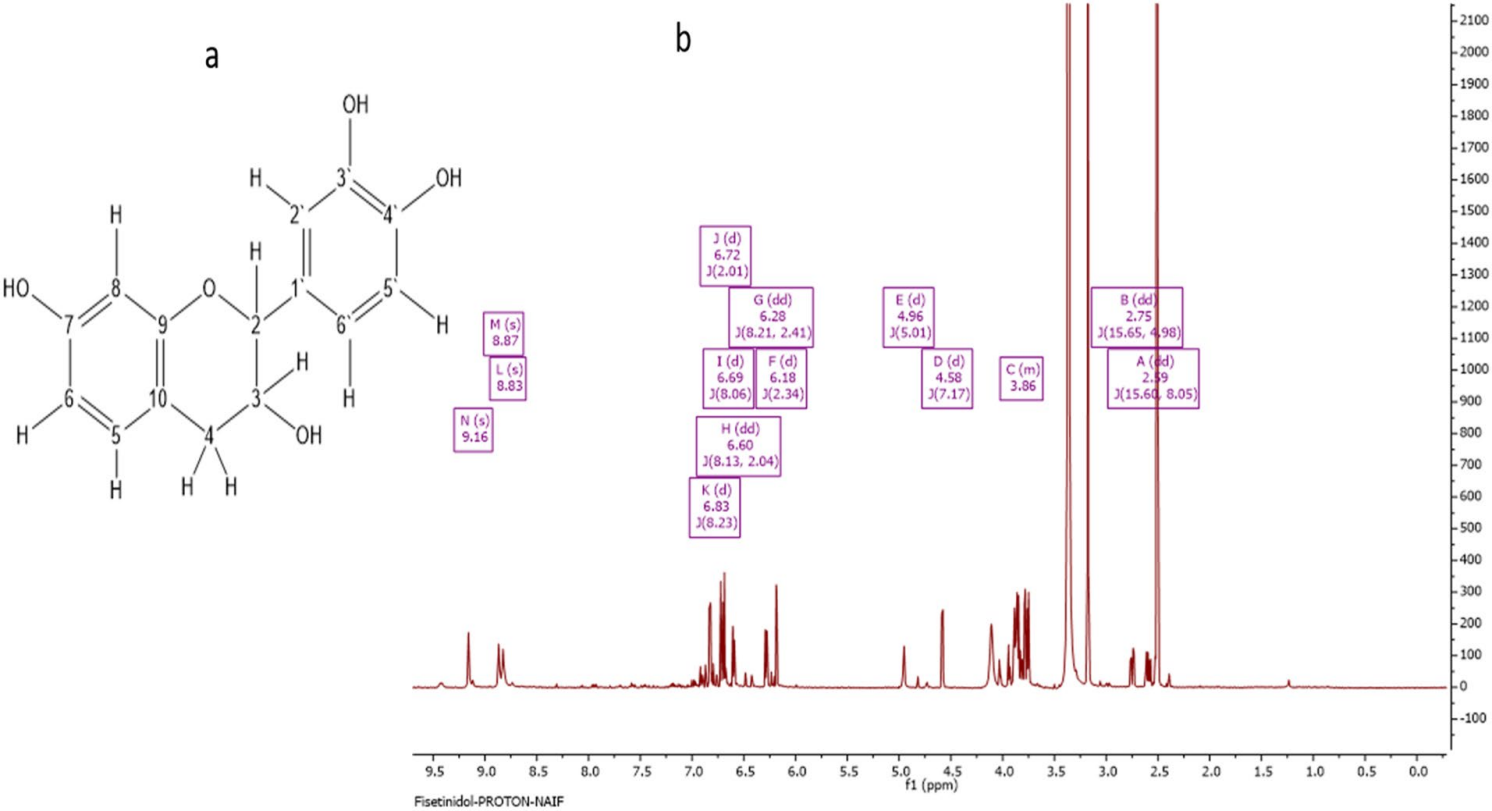
Characterization of S-6-7 as fisetinidol. (**a**) Structure of fisetinidol; (**b**) NMR spectrum of fisetinidol.

The complete characterization and structural elucidation of the S-6-7 fraction is depicted in Fig. 3. Analysis by LC–HRESI–MS gave a molecular ion at m/z 273.08 [M–H]–; a calculated mass of C_15_H_13_O_5_ = 273.08 (Fig. 3a), and the optical rotation had a value of − 5.9° (*c* = 0.085, MeOH). Its ^1^H NMR spectrum showed two sets of aromatic ABX spin systems. The first set was at δ_H_ ppm 6.83 (d, *J* = 8.23 Hz), 6.28 (dd, *J* = 8.21, 2.41), and 6.18 (d, *J* = 2.34). The second set of aromatic ABX protons was at 6.72 (d, *J* = 2.01), 6.69 (d, *J* = 8.06), and 6.60 (dd, *J* = 8.13, 2.04). It also showed two oxymethine protons at 4.58 (d, *J* = 7.17) and 3.86 (m). There were also two methylene protons at 2.75 (dd, *J* = 15.65, 4.98) and 2.59 (dd, *J* = 15.60, 8.05). Finally, there three phenolic protons at 9.16 (s), 8.87 (s), and 8.83 (s). The ^13^C spectrum showed 15 signals consisting of six aromatic CH carbons and two oxymethine carbons at δ_C_ ppm 81.65 and 66.79. The rest of the carbon signals were for a methylene carbon at 32.71, four phenolic carbon atoms, and two aromatic quaternary carbons at 111.52 and 131.02 ppm. The absence of a hydrogen-bonded -OH proton around 12–13 ppm and a carbonyl signal between 170 and 220 ppm indicates the compound is not a flavone but a flavan derivative. This was confirmed from its 2D NMR spectrum as long-range HMBC correlations from H-2 to C-9, C-12, C-16, C-4, C-11, and C-3 were identified, while correlations from H-4 to C-10, C-5, and C-9 were identified. The 7-OH gave correlations to C-6, C-7, and C-8. Other correlations from the HMQC and COSY confirmed the structure of the compound and the carbon and proton chemical shifts (Supplementary, Fig. S1a–d). The chemical structures of the main compounds identified are shown in Fig. 3a. This compound was identified as fisetinidol.

### Characterization of S-6-13 as ferulic acid

CC and then SEC was performed to isolate S-6-13 from the ethanol it was extracted in.

**Figure 4:**
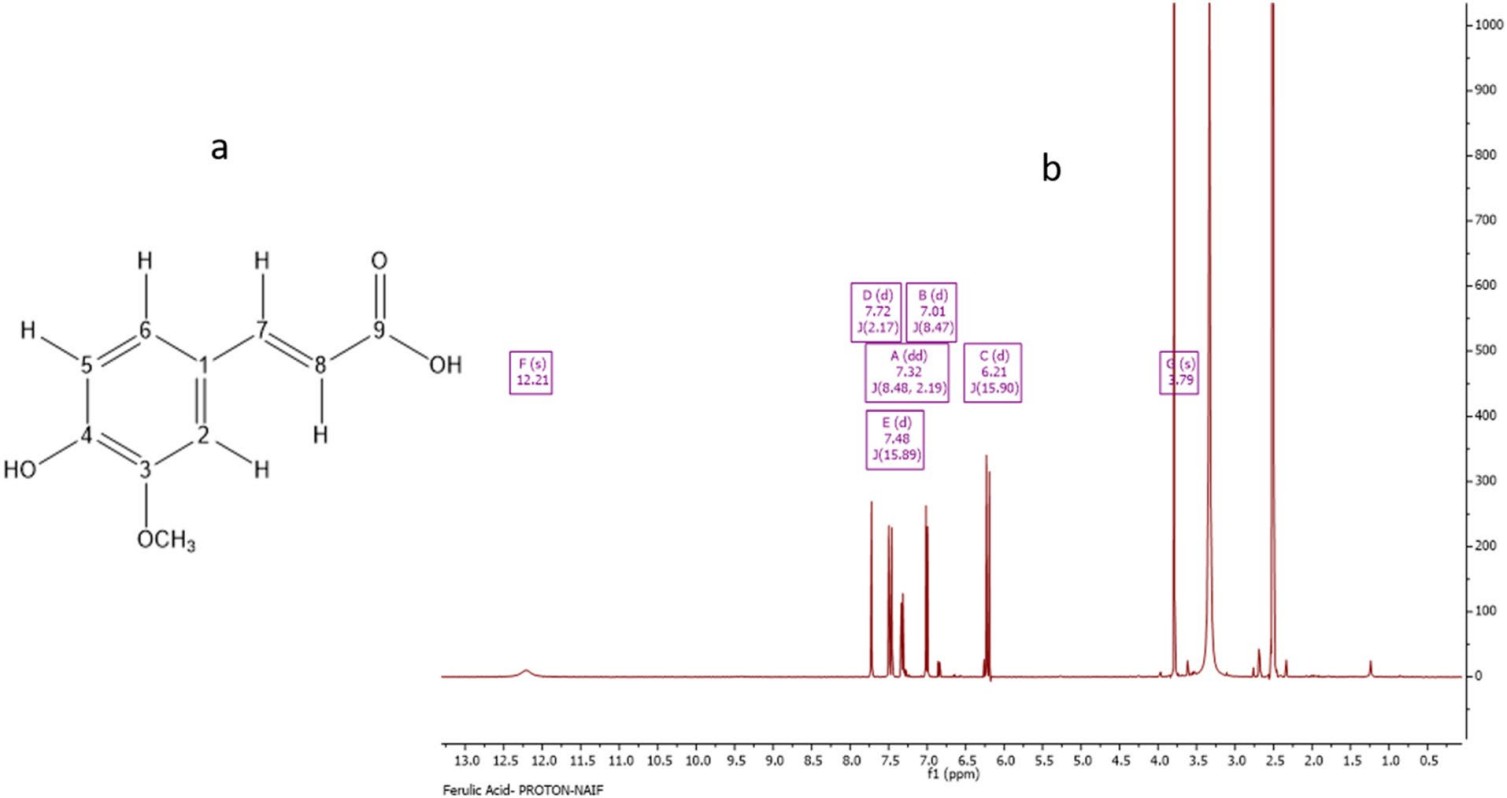
Characterization of S-6-13 as ferulic acid. (**a**) Structure of ferulic acid; (**b**) ^1^H NMR spectrum (400 MHz) of ferulic acid (S-6-13).

Analysis by LC–HRESI–MS yielded a molecular ion at m/z 193.05 [M–H]–; a calculated mass of C_10_H_9_O_4_ = 273.08 (Fig. 4a). Figure 4b depicts the ^1^H-NMR exhibiting two doublets at δ_H_ 6.21 (1H, d, *J* = 15.90, H-8) and 7.48 ppm (1H, d, *J* = 15.89, H-7). Three aromatic proton signals at 7.32 (1H, dd, *J* = 8.48, 2.19, H-6), 7.01 (1H, d, *J* = 8.47, H-5), and 7.72 (1H, d, *J* = 2.17, H-2). It also showed a methoxy signal at 3.79 (3H, s, 5-OCH_3_). The ^13^C-NMR spectrum showed a deshielded signal at δ_C_ 168.28 ppm for a carboxylic acid carbonyl group (C-9), two olefinic CH at 116.86, 144.36, three aromatic CH at 112.92, 119.9, and 125.11, two oxygenated aromatic carbons at 143.29 and 153.26, a quaternary carbon at 126.72, and a methoxy carbon at 56.16 ppm. This was confirmed from its 2D NMR spectrum as long-range HMBC correlations from H-2 identified as C-4, C-6, and C-7, while correlations from H-5 identified as C-1 and C-3. Also, the correlations from H-6 were identified as C-2, C-4, and C-7, while the correlations from H-7 were identified as C-2, C-6, and C-9. HMBC correlations from H-8 were identified as C-1 and C-9. Other correlations from HMQC and COSY confirmed the compound’s structure and the carbon and proton chemical shifts (Supplementary, Fig. S2a,b). The compound was identified as ferulic acid.

### Biological evaluation of Saudi propolis sample against trypanosomes (*T. brucei S427* strain)

Crude, fractions, and pure compounds (fisetinidol and ferulic acid) extracted from Saudi propolis samples were tested against *T. brucei.* Pentamidine and Diminazen were used as drug controls with a minimum inhibitory concentration (MIC) score of 0.0030 and 0.0313 µg/ml, respectively, as shown in Table 2. The S-6 fraction demonstrated the highest anti-trypanosomal activity at 2.4 µg/ml MIC, followed by Saudi crude with 4.6 µg/ml MIC, where fisetinidol and ferulic acid had MICs of 14.7 and 39.9 µg/ml, respectively.

**Table 2:** Drug Sensitivity assay of Saudi propolis sample and its fractions on *T. brucei* S427 WT.

Sample code	Mean (µg/ml)	SD
Saudi crude	4.6	0.21
S-6 fraction	2.4	0.24
Fisetinidol	14.7	0.27
Ferulic acid	39.9	1.19
Pentamidine(µM)	0.0030	0.0007
Diminazen(µM)	0.0313	0.0065

### Anticancer effect of Saudi propolis

The cytotoxicity assay of the Saudi propolis sample and its fractions on U937 cells is shown in Table 3.

**Table 3:** Cytotoxicity assay of Saudi propolis sample and its fractions on U937 cells.

Sample code	IC_50_ mean (µg/ml)	SD	%RSD
Saudi crude	129.1	6.85	5.31
S-6 fraction	58.7	2.98	5.08
Fisetinidol	256.9	30.19	11.75
Ferulic acid	87.7	5.74	6.54
Pentamidine(µM)	13.3167	1.0148	7.6202
Diminazen(µM)	29.5767	2.1704	7.3381

The S-6 fractions exhibited the highest cytotoxic activity against U937 cells with an IC_50_ of 58.7 µg/ml followed by ferulic acid with an IC_50_ of 87.7 µg/ml, indicating that the cytotoxic effect of propolis might be attributed to the presence of ferulic acid.

## Discussion

The present study demonstrated the anti-trypanosomal and anticancer properties of various propolis extracts crude and pure fractions, and the highest activity was found in the S-6 fractions. The chemical profiling by GC–MS and ^1^H NMR spectra demonstrated flavonoids and phenols in propolis extracts. All crude extracts, fractions, and isolated compounds (ferulic acid and fisetinidol) tested against *Trypanosoma brucei* (S427 WT) and for cytotoxicity against U937 human leukemia cells yielded satisfactory results. Among the samples tested, the highest antiparasitic and anticancer effect was observed in S-6 fractions due to the combined effect of ferulic acid, fisetinidol, and other various compounds.

The NMR spectral data (Fig. 3b) from the S-6-7 fraction was in agreement with previous reports^25^. The compound identified was fisetinidol, indicating that this compound is typical of Saudi propolis. Similarly, the S-6-13 fraction was identified as ferulic acid, and its NMR spectral data were in agreement with earlier findings^26,27^. The compounds detected are known constituents of propolis like ferulic acid. Yet, the presence of fesitinidol is comparatively rare. Propolis has been reported to contain over 300 compounds, but not all compounds are related to its biological effects. Standardization of propolis samples from different geographical areas with different biological effects has not been done. The occurrence of ferulic acid with the elemental composition of C_10_H_9_O_4_ in the propolis extract in the current study is consistent with findings from El-Mawla and Osman^28^. Several studies have reported the potential biological effects of these compounds against bacteria, cancer^26–29^, and leishmaniasis^30^ from different geographical regions. Reports on the biological activity of propolis are inconsistent, and little is known about the biological activities of Saudi propolis.

*Trypanosoma brucei* is a branching, flagellated protozoan parasite that causes sleeping sickness in humans and nagana in cattle in sub-Saharan Africa, where it can be devastating^31^. The current WHO estimate indicates that about 30,000 people are infected per year, and over 70 million people are at risk of infection^32^. Livestock in the Middle east is at higher risk of infection and heavy loss of cattle. Given the severity, the anti-trypanosomal property of the propolis was investigated. The observed anti-trypanosomal activity (Table 2) was in line with Khalil et al.^33^ and Alotaibi et al.^34^. Contrarily, Almutairi et al. found fisetinidol extracted from Saudi propolis to be inactive against trypanosomes and bacteria^25^. The main component of the S-6 fraction exhibiting the highest antiparasitic effect identified in this study was fisetinidol. The observed decrease in the anti-trypanosomal activity of isolated compounds compared to the mixed fraction is in line with reports of Omar et al.^35^.

Chemotherapy is one of the major therapeutic approaches for treating benign and metastasized cancer; however it has some limitations. Hence, it is extremely important to design new, natural, and efficient drugs. Propolis has been shown to possess beneficial activities for human health since ancient times. Importantly, propolis was observed to play a key role in tumor regulation via its cytotoxic effects. Among the three tested samples S-6-7, ferulic acid, and fisetinidol, the S-6-7 fraction had the highest cytotoxicity while fisetinidol had the least. Between the two pure compounds, the cytotoxic activity of ferulic acid was much more pronounced. Thus, the cytotoxic activity of S-6-7 could be attributed to the presence of ferulic acid. Based on the above observations, ferulic acid was considered to enhance the cytotoxic property of the S-6-7 fraction. The decrease in the isolated fraction’s anticancer activity compared to the mixed S-6 fraction suggests a synergistic anticancer effect between the compounds. Nevertheless, there was significant anticancer property demonstrated by propolis. The cytotoxicity against cancer cells was in accordance with previous reports^35–37^.

As previously stated, the isolated compounds demonstrating lower activity than the mixed fraction or the crude extract could be reasoned as follows; retention of the active compound in the column; instability of the active compound in the conditions used in the isolation process; the distribution of the majority of active constituents over different fractions; or synergistic interaction between several compounds may be the source of the extract effects^38^. In line with current report, many studies have revealed that the observed effects might be the result of synergistic action of its complex constituents and propolis exhibiting higher activity than isolated compounds^39,40^. Based on the current investigation’s data, Saudi propolis emerged to possess biological activity as a promising antiparasitic and anticancer agent.

## Methods

### Collection and preparation of the propolis sample

The Saudi propolis sample was collected from the Rijal Alma’a village, Asir region (N 2015840.65, E 211634.87), South-west of Saudi Arabia during summer. The samples were stored at room temperature, away from light and humidity, until further analysis.

### Processing and ethanolic extraction of Propolis samples

Raw propolis was macroscopically screened to remove impurities (e.g., pollen, wood, and dead bees) prior to extraction. A mortar and pestle were used to fragment the samples of propolis. For extraction, 5 ml of ethanol was added to 50 mg of propolis sample and sonicated for 180 min, and reextracted thrice. A syringe filter (Acrodisc 0.45 μm) was used to filter the samples, and a nitrogen flow was used for drying the filtered solution. To obtain pure components, further fractionation, and purification of raw propolis was achieved by CC and SEC. An appropriate quantity of absolute ethanol (100 ml/g) was added to raw propolis and sonicated for an hour to obtain ethanol-based extract for fractionation. An appropriate quantity of ethanol was subsequently used to filter and re-extract two times, with subsequent filtering every time. After the extracts were merged, a rotary evaporator was used to evaporate and dry the solvent, followed by weighing. An extraction yield of 12.8869 g was obtained. A small quantity (1 ml) of ethyl acetate was used to re-dissolve the residue (2 mg) completely, followed by sonication to stimulate the dissolution of the residue. The extracted solution was poured into empty weighed vials and labeled. Crude propolis samples and purified fractions were analyzed by LC–MS, HPLC in association with a range of detectors, including ELSD, UV, and high resolution mass spectrometry, as well as NMR spectroscopy.

### Isolation and characterization of pure compounds

#### CC and SEC

Isolation and purification of compounds from the crude extract were achieved by employing CC and SEC^41,42^. Around 6.25 g of the ethanol-based extract of Saudi propolis was subjected to CC and gradient elution with solvents of different polarities. Silica gel 60 with a mesh size of 200–425 μm was used. A rotatory evaporator was used to collect and concentrate the fractions, which were then aggregated via HPLC–UV–ELSD analysis based on similar chemical profiles. For SEC, Sephadex LH 20 was used for column packing, and elution was performed in an isocratic manner with methanol.

#### LC–MS

LC/MS was carried out using a Dionex 3000 UHPLC pump coupled to an Exactive (Orbitrap) Mass spectrometer, Thermo Fisher Scientific (Bremen, Germany)**.** Crude samples and purified compounds were prepared at 1 mg/ml in methanol prior to LC–MS. A reverse-phase 5 µm C18 column (4.6 × 150 mm) (Hypersil, Thermo) was used, and the elution was carried out using a gradient at a flow rate of 0.3 ml/min, with 0.1% v/v formic acid in water and 0.1% v/v formic acid in acetonitrile (the A and B solvents) making up the mobile phase. The ESI interface in negative ionization permitted identification of [M-H] -. The spray voltage for the capillary and cone were − 4.0 kV and 35 V, respectively. The flow rate of the sheath gas and auxiliary gas were 50 and 15 arbitrary units, respectively. The ion transfer capillary had a temperature of 275 °C, and m/z between 100 and 1500 provided the full scan data^43^. The sample data were acquired and processed with Xcalibur software (Thermo Fisher Corporation, Hemel Hempstead, UK).

#### HPLC–UV–ELSD

A 1 mg/ml solution was produced for every sample dried under nitrogen in 1 ml of the mobile phase LC gradient's initial composition. A reverse-phase 5 µm C18 column (4.6 × 150 mm) (Hypersil, Thermo) was employed for separation purposes, with water and acetonitrile (the A and B solvents) in the mobile phase. An Agilent 1100 system (Agilent Technologies, Germany) consisting of a quaternary pump, a diode array UV detector (set to monitor 290 and 320 nm wavelengths), and an ELSD (SEDEX75 SEDERE, France) constituted the used HPLC–UV–ELSD equipment. Data were collected and processed using Clarity software (Data Apex)^44^.

### Structural elucidation by nuclear magnetic resonance spectroscopy (NMR)

The ^1^H, ^13^C, and 2D ^1^H, ^1^H-COSY, ^13^C-1H HSQC, and HMBC NMR spectra were obtained using a JEOL-LA 400 FT-NMR spectrometer with tetramethylsilane (TMS) as an internal standard^45,46^. Based on the solubility of the compounds, preparation of the sample solutions involved the use of deuterated solvents like CDCl3 and DMSO—d6 that have residual proton shifts and carbon shifts. Around 500–600 μl of a suitable solvent was used to dissolve 10 mg of every sample, which was then poured into a typical 5 × 178 mm NMR tube to a depth of around 4 cm. MestReNova software 8.1.2 (Mestrelab Research, A Coruña, Spain) was used to process the NMR spectroscopic data, and the structures of the compounds were illustrated using ChemBioDraw Ultra, Version 14 (PerkinElmer, Yokohama, Japan).

### Cell culture and medium preparation

The U937 cells (a human monocytes cell line), were cultured in the RPMI 1640 medium (500 ml), which was supplemented with 1% penicillin and streptomycin (v/v), 1% l-glutamine (v/v), and 5% FCS (v/v). Cells were sub-cultured every 2–4 days and maintained at 37 °C in 5% CO_2_.

### Cell viability assay

The U937 cells were seeded at 1 × 10^5^ cells/ml in 96-well plates. The cells were counted manually using a haemocytometer under a microscope. The cells were added and the plate incubated for 24 h at 37 °C in 5% CO_2_. The samples (the crude, fractions, and purified compounds) were prepared at 8 different concentrations in another 96 well plate to prevent disturbing the cells during mixing since U937 cells do not adhere. The highest concentration started from 200 μg/ml and a serial 1:2 dilution was carried out until the concentration was 1.56 μg/ml (n = 3). The samples were then transferred (100 μl) to the cultured cells using a multichannel pipette and placed in the incubator for 24 h. 10% (v/v) DMSO was added to serve as a positive control (to kill the cells completely). The cells in medium alone were used as a negative control and 0.5% (v/v) DMSO (the solvent concentration in the samples) was tested as well. The plate was then incubated at 37 °C in 5% CO_2_ for 24 h. After incubation, the resazurin indicator (Alamar blue) was added at a final concentration of 10% and incubated for a further 24 h. Fluorescence was read using a Wallac Victor 2 microplate reader (λEx/EM: 560/590 nm). Cell viability was then calculated for each well as the percentage of fluorescence in the test samples relative to the values of the negative controls. The resulting data were analyzed with GraphPad Prism 5 to obtain dose–response curves for each sample and their corresponding IC_50_ values.

### Anti-trypanosomal assay

Crude extracts, fractions, and isolated compounds were tested against the bloodstream form of wild-type *Trypanosoma brucei* (S427) in vitro. *Trypanosoma brucei*, Lister 427 were cultured as previously described^47^. The anti-trypanosomal tests were carried out using an Alamar blue assay^48^. This assay is based on viable cells metabolizing the blue resazurin dye to resorufin, which is pink and fluorescent. It was performed using stock solutions of the samples prepared with a 20 mg/ml concentration in 100% DMSO with subsequent dilution of DMSO concentration to 0.1%. The assays were performed using (1:1) serial dilution of test compounds in Hirumi’s Modified Iscove’s medium 9 (HMI-9), where 100 μl of each compound or fraction was doubling diluted over one row in the 96-well plate, (starting from 200 µg/ml as the top concentration until 0.19 µg/ml) ensuring an optimally defined 50% Effective Concentration (EC_50_) after plotting the reading to a sigmoid curve with a variable slope using GraphPad Prism software. A 100 μl of trypanosome suspension was eventually added to each well plate at a seeding density of 2 × 10^5^ cells followed by an incubation period of 48 h at 37 °C in a 5% CO_2_ humidified incubator. Before adding the resazurin dye and further incubation for 24 h under the same conditions, fluorescence was determined in a FLUOstar Optima (BMG Labtech) at wavelengths of 544 nm and 620 nm for excitation and emission, respectively.

## Supplementary Information

Supplementary Information 1.

Supplementary Information 2.

Supplementary Information 3.
