# Supplementary material for: Chemical characterization of Saudi propolis and its antiparasitic and anticancer properties
Source: Sci Rep. 2021 Mar 8;11:5390. doi: 10.1038/s41598-021-84717-5 (PMC7970881; doi:10.1038/s41598-021-84717-5)
Supplement: Supplementary file 1 — Supplementary Information 1. [file 41598_2021_84717_MOESM1_ESM.doc]

**Chemical characterization of Saudi Propolis and its antiparasitic and anticancer properties**

Samyah Alanazi^1*^, Naif Alenzi^2^, Fouza Alenazi^2^, Hajera Tabassum^1^,David Watson^3^

^1^Department of Clinical Laboratory Sciences, College of Applied Medical Sciences, King Saud University, Riyadh, Saudi Arabia.

^2^Research and Laboratories Sector, National Drug and Cosmetic Control Laboratories (NDCCL), Saudi Food and Drug Authority, Riyadh, Saudi Arabia.

^3^Strathclyde Institute of Pharmacy and Biomedical Sciences, University of Strathclyde,United Kingdom.

**Corresponding author**

**Dr. Naif D. Alenzi**

Research and Laboratories Sector,

National Drug and Cosmetic Control Laboratories (NDCCL),

Saudi Food and Drug Authority,

Riyadh, Saudi Arabia

Email: Ndenzi@sfda.gov.sa

**Supplementary Figures**

**Figure S1. Spectral data on identification of fisetinidol**

1. Full DEPTq 135 ^13^C NMR spectrum (100 MHz) of fisetinidol (S-6-7); b) COSY spectrum (400 MHz) of fisetinidol (S-6-7); c) COSY correlations NMR spectra of fisetinidol; d) LC-UV-ELSD chromatogram of fisetinidol purified from SEC.

**Figure S2. Spectral data on identification of ferulic acid**

a) ^13^C NMR spectrum (100 MHz) of ferulic acid (S-6-13); b) COSY spectrum (400 MHz) of ferulic acid (S-6-13); c) LC-UV-ELSD chromatogram of ferulic acid purified from SEC.
