# Supplementary figures and images for: Chemical characterization of Saudi propolis and its antiparasitic and anticancer properties
Source: Sci Rep. 2021 Mar 8;11:5390. doi: 10.1038/s41598-021-84717-5 (PMC7970881; doi:10.1038/s41598-021-84717-5)

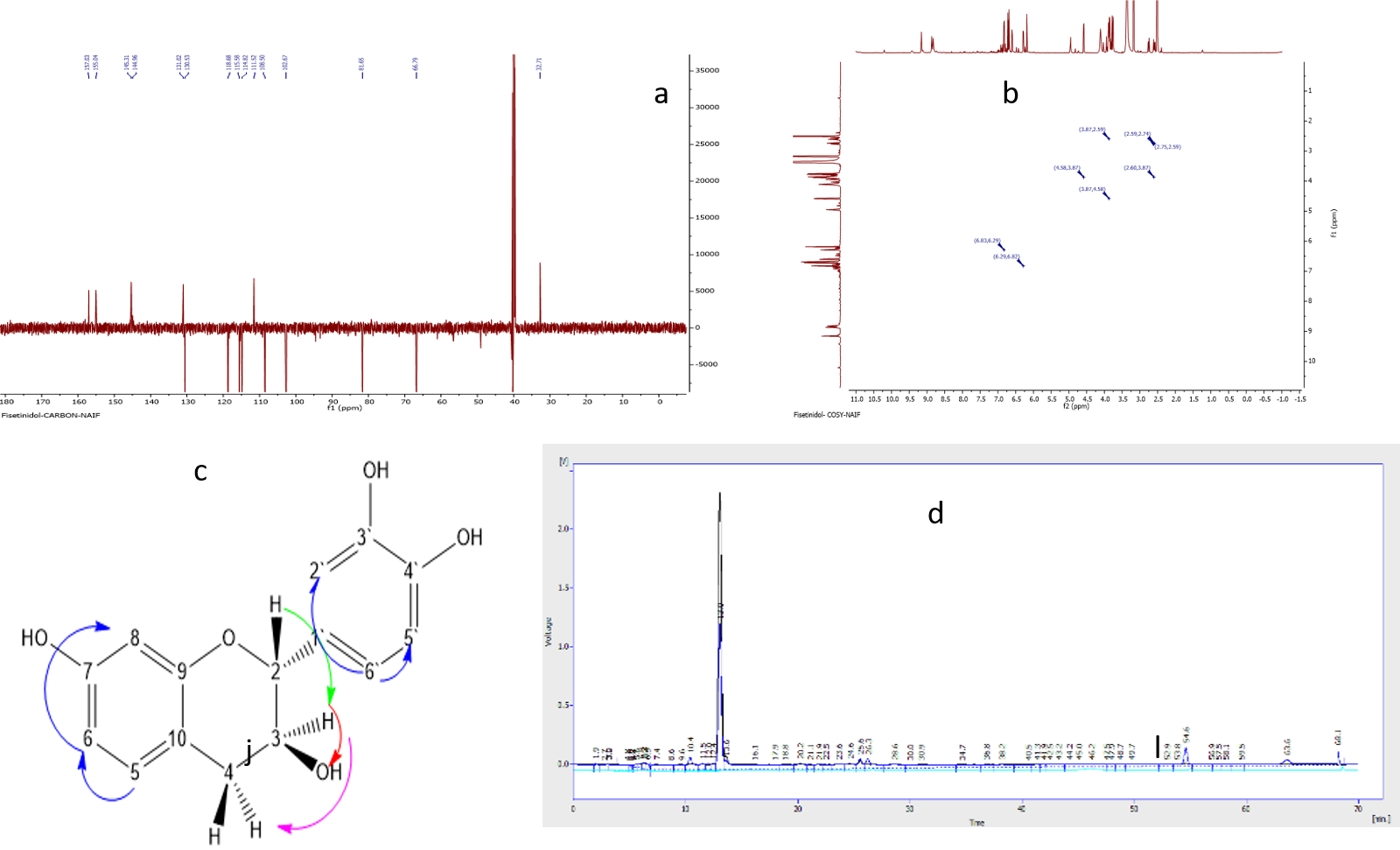

Supplement: Supplementary file 2 — Supplementary Information 2. [file 41598_2021_84717_MOESM2_ESM.tif]

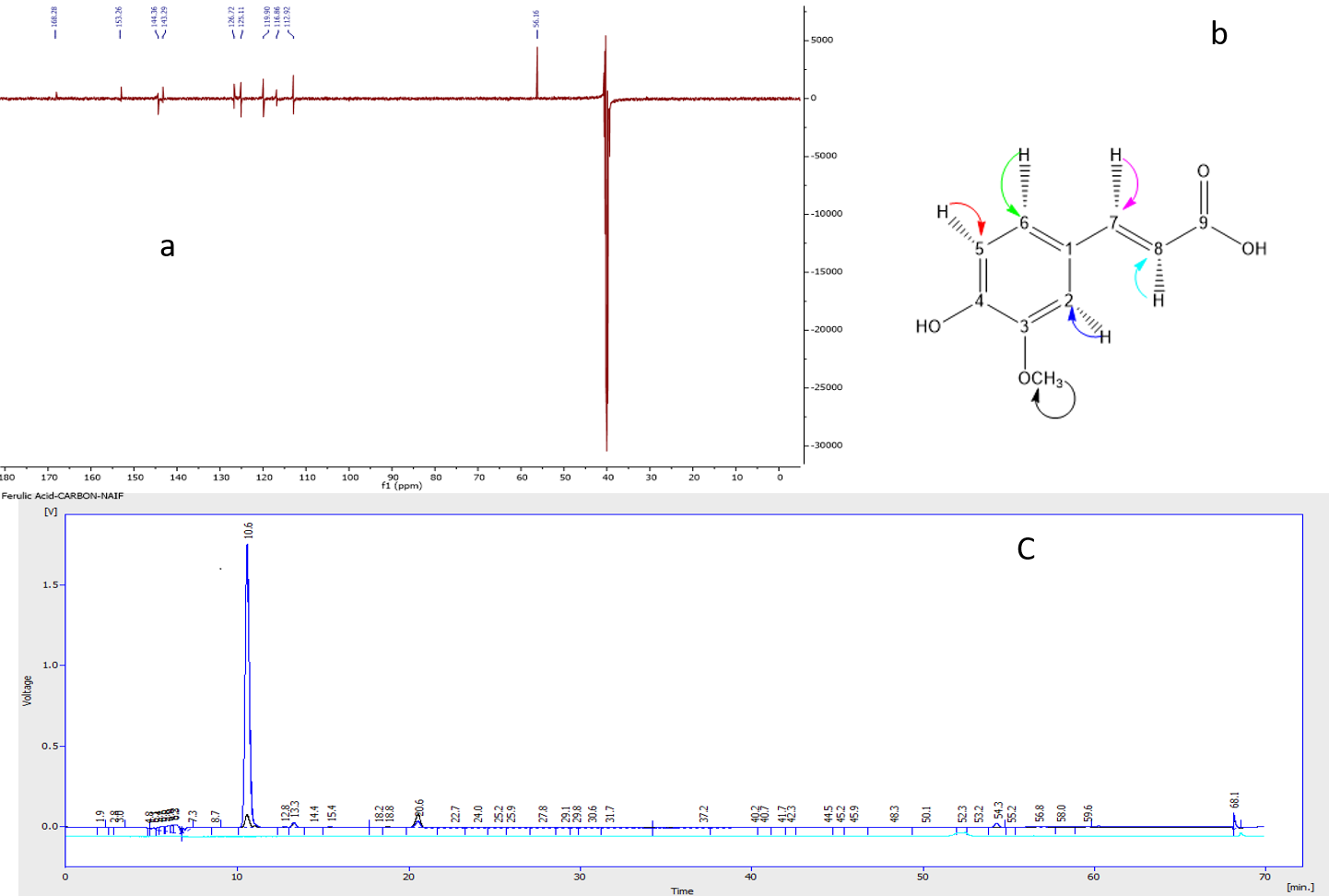

Supplement: Supplementary file 3 — Supplementary Information 3. [file 41598_2021_84717_MOESM3_ESM.tif]
